# Central Nervous System Injury – A Newly Observed Bystander Effect of Radiation

**DOI:** 10.1371/journal.pone.0163233

**Published:** 2016-09-30

**Authors:** Caitlin Feiock, Masashi Yagi, Adam Maidman, Aaron Rendahl, Susanta Hui, Davis Seelig

**Affiliations:** 1 Department of Veterinary Clinical Sciences, College of Veterinary Medicine, University of Minnesota, St. Paul, Minnesota, United States of America; 2 Department of Therapeutic Radiology, University of Minnesota, Minneapolis, Minnesota, United States of America; 3 School of Statistics, University of Minnesota, Minneapolis, Minnesota, United States of America; Rutgers University, UNITED STATES

## Abstract

The unintended side effects of cancer treatment are increasing recognized. Among these is a syndrome of long-term neurocognitive dysfunction called cancer/chemotherapy related cognitive impairment. To date, all studies examining the cognitive impact of cancer treatment have emphasized chemotherapy. Radiation-induced bystander effects have been described in cell culture and, to a limited extent, in rodent model systems. The purpose of this study was to examine, for the first time, the impact of non-brain directed radiation therapy on the brain in order to elucidate its potential relationship with cancer/chemotherapy related cognitive impairment. To address this objective, female BALB/c mice received either a single 16 gray fraction of ionizing radiation to the right hind limb or three doses of methotrexate, once per week for three consecutive weeks. Mice were sacrificed either 3 or 30 days post-treatment and brain injury was determined via quantification of activated astrocytes and microglia. To characterize the effects of non-brain directed radiation on brain glucose metabolism, mice were evaluated by fluorodeoxygluocose positron emission tomography. A single fraction of 16 gray radiation resulted in global decreases in brain glucose metabolism, a significant increase in the number of activated astrocytes and microglia, and increased TNF-α expression, all of which lasted up to 30 days post-treatment. This inflammatory response following radiation therapy was statistically indistinguishable from the neuroinflammation observed following methotrexate administration. In conclusion, non-brain directed radiation was sufficient to cause significant brain bystander injury as reflected by multifocal hypometabolism and persistent neuroinflammation. These findings suggest that radiation induces significant brain bystander effects distant from the irradiated cells and tissues. These effects may contribute to the development of cognitive dysfunction in treated human cancer patients and warrant further study.

## Introduction

Survivorship issues are increasingly recognized as untoward consequences of improving successes in cancer treatment. Chief among these is a syndrome of cognitive impairment known as “chemobrain” or “chemofog.” While originally thought to be solely psychological in origin, there is increasing acceptance that chemobrain reflects true neurobiological side effects of cancer treatment. This syndrome, now referred to as “cancer/chemotherapy related cognitive impairment (CRCI),” is clinically characterized by loss of memory, concentration, and executive function. CRCI affects up to 75% of treated cancer patients and can persist for 5–10 years post cessation of treatment [1,2].

Despite an increasing recognition of its incidence and its increasing appearance in the scientific literature, the mechanisms underlying CRCI are unknown. Current research suggests that CRCI principally results from either the direct neurotoxic effects of chemotherapy, the spread of reactive oxygen species (ROS) and inflammatory cytokines from peripheral tissues to the CNS, or alterations in brain blood flow [3]. Microscopically, CRCI is characterized by neuroinflammation, decreased blood vessel density, and decreased neurogenesis [3]. In chemotherapy-treated mice, the neuroinflammatory phenotype is reflected by increased numbers of microglia cells (i.e. microgliosis) or, less commonly, increased numbers of astrocytes (i.e. astrocytosis) [3–5]. Despite these clinical, radiological, and microscopic observations, a complete mechanistic narrative of CRCI has yet to be told and the contributions of non-chemotherapy treatments have yet to be characterized.

Ionizing radiation therapy (RT) is a mainstay of modern cancer treatment, as between one-half and two-thirds of all newly diagnosed cancer patients receive RT at some point during treatment (2), including 56% of breast and prostate cancer patients [6,7] and 18% of non-Hodgkin Lymphoma patients [8]. Although it was long believed that the biological effects of ionizing radiation were restricted to cells and tissues within the treatment field, this view been challenged with the identification of radiation-induced bystander effects (RIBEs). These bystander effects, defined as biological phenomena exhibited by neighboring and distant un-irradiated cells due to signals received from irradiated cells, encompass both detrimental (chromosomal aberrations, increased mutagenesis, and inflammatory mediator production) and beneficial (shrinkage out-of- field metastases) phenomena [9]. In rodents, the anatomic scope of bystander injury has been illustrated through studies demonstrating cell injury and increased mutagenesis in the shielded lung and brain of irradiated mice [10,11]. However, despite these reports, the scope of the physiological consequences of RIBEs on the CNS has yet to be fully documented in a robust model system.

The syndrome of CRCI is not restricted to patients treated with chemotherapy alone as up to 67% of patients treated with non-brain directed radiation therapy (NBRT) for a variety of malignancies experience self-reported memory deficits and concentration impairments that persist for several years after RT [12–14]. Similarly, limited clinical studies in breast cancer patients reveal that NBRT is capable of inducing deficits in attention, complex cognition, and attention that emerge 6 months post-treatment and persist for up to 3 years [15,16]. Although there are an estimated 4.5 million people affected by CRCI [8], most of which will have received some form of RT, the impact of NBRT on the brain has yet to be fully examined. In light of the increasing appreciation of radiation’s bystander effects, the frequency of CRCI, and the ubiquity of RT as a cancer treatment, we hypothesize that NBRT has the potential to exert brain bystander effects which might ultimately contribute to the development of cognitive impairment in cancer patients.

To address to the potential contribution of NBRT to the development of CRCI, we investigated the *in vivo* bystander effects of NBRT on the brains of mice receiving hind-limb targeted ionizing radiation. To characterize the biological and pathologic consequences of NBRT on the brain, we evaluated brain glucose metabolism and glial cell activation. To characterize the longitudinal effects of NBRT on the brain, neuropathology was evaluated at days 3 and 30 post treatment. Additionally, we sought to contextualize our findings by evaluating the severity of NBRT induced neuropathology as compared to methotrexate, a chemotherapeutic agent known to contribute to the development of CRCI in both humans and mice [17]. In these studies we revealed that mice administered NBRT demonstrate global brain glucose hypometabolism as well as acute and persistent multifocal neuroinflammation. Moreover, we demonstrate that the neuroinflammatory effects of NBRT are statistically indistinguishable from methotrexate treatment, which supports the potential cognitive impact of non-brain directed radiation therapy.

## Methods and Materials

### Animals and Treatments

**Fig 1:**
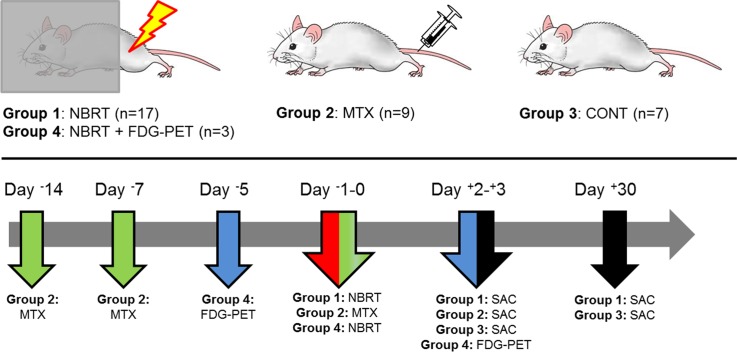
Summary of Experimental Design. Green arrows indicate methotrexate (MTX) injection. Blue arrows indicate FDG-PET. Red arrows indicate non-brain directed radiation therapy (NBRT). Sham-treated mice are indicated by CONT. Black arrows indicate sacrifice. Multi-colored arrows indicate multiple procedures occurring within the indicated time frame.

All experimental procedures were approved by the Institutional Animal Care and Use Committee of the University of Minnesota. Study animals consisted of (n = 36) sixteen-to-eighteen week old, female, BALB/c mice (Harlan Sprague Dawley, Inc., Madison, WI). Mice were randomly assigned into one of three treatment groups, namely: mice administered ionizing radiation (NBRT, n = 17), mice administered methotrexate chemotherapy (MTX, n = 9), and untreated controls (CONT, n = 7). To examine the effects of NBRT on brain glucose metabolism, a separate cohort of mice (n = 3) were serially evaluated using ^18^F-fluoro-2-deoxy-D-glucose positron emission tomography ([^18^F]FDG-PET) prior to, and 3 days following, irradiation (NBRT+FDG). The serial, intra-individual evaluation of [^18^F]FDG-PET metabolism allows each mouse to serve as its own non-irradiated control and, consistent with previous mouse brain PET imaging studies, accounts for potential animal-specific responses to NBRT [18]. The study design is summarized in Fig 1.

NBRT mice were administered a single 16 Gy fraction of orthovoltage ionizing radiation to the right hind limb using an XRad 320 Biological Irradiator (Precision X-Ray, Inc., North Branford, CT). This radiation dose was chosen based upon previous work confirming its clinical relevance in mice models [19]. During NBRT, mice were anesthetized (2.0 ml ketamine/0.2 ml xylazine [100 mg/ml] in sterile saline) and shielded using a custom 4 mm thick lead shield, leaving only the right hind limb exposed. The MTX group received 3 intraperitoneal injections of methotrexate dissolved in saline (0.75 mg/kg) administered once per week for 3 consecutive weeks. The dose of MTX was chosen as it has been shown to be clinically relevant in previous rodent studies [20–22]. To control for the potentially confounding effects of anesthesia, CONT mice were anesthetized in a manner identical to the NBRT mice.

To characterize the sequential effects of NBRT, irradiated and control mice were sacrificed at day 3 (n = 8 NBRT, n = 3 NBRT+FDG and n = 3 CONT) and day 30 (n = 9 NBRT and n = 4 CONT) post-irradiation. The MTX mice were sacrificed 3 days after the final dose was administered (17 days post first treatment). Mice were sacrificed in accordance with the American Veterinary Medical Association guidelines using carbon dioxide asphyxiation. At euthanasia, brains were removed and bisected. One half was fixed in 10% neutral buffered formalin (NBF) and the remaining half was frozen at -80°C. During the study, all mice were housed in a standard vivarium and allowed *ad libitum* access to regular mouse diet and water.

### FDG-PET Imaging and Image Analysis

The [^18^F]FDG-PET imaging was performed as previously described [23]. In brief, mice were administered [^18^F]FDG (18.9 ± 1.6 MBq per 100 μl solution) intravenously via the tail vein and were imaged using an Inveon^TM^ PET system (Siemens Medical Solutions, Knoxville, TN) during a 30-minute acquisition using the manufacturer’s recommended acquisition settings. During acquisition, mice were maintained under 1.5–3% isoflurane anesthesia in a 1 L/min O_2_ nose cone on a warming pad and were monitored with a respiration monitor (BioVet, Spin Systems Pty Ltd., Brisbane, Australia). Inveon acquisition workplace software (IAW, version 1.5.0.28; Siemens Medical Solutions) was used for PET image reconstruction, as described previously [23]. PMOD software, version 3.4 build 6 (PMOD Technologies, Zurich, Switzerland) was used for scan analyses. Regions of interest (ROI) were identified semi-automatically using the segmentation feature based on a 3D region-growing algorithm and manual modification to minimize variations. Fourteen ROI were selected: olfactory bulb, striatum, basal forebrain and septum, cortex, hippocampus, thalamus, hypothalamus, amygdala, cerebellum, brainstem, superior colliculi, inferior colliculi, midbrain, and cingulum. The uptake values were normalized by blood, as previously described [23]. In light of the exploratory nature of this study, [^18^F]FDG-PET analysis is only available for the NBRT and CONT mice [23].

### Immunohistochemistry

For the immunohistochemical (IHC) studies, five micron tissue sections from formalin-fixed, paraffin-embedded brains were immunostained for activated astrocytes (rabbit polyclonal anti-GFAP; Abcam, Cambridge, MA), activated microglia (rabbit polyclonal anti-Iba1; Abcam, Cambridge, MA). A subset of animals were immunostained for tumor necrosis factor-alpha (TNF-α) (rabbit polyclonal anti-TNF-α; Abcam, Cambridge, MA). Tissue sections were deparaffinized in xylene, rehydrated through graded alcohols, and subjected to antigen retrieval at 80°C for 1 hour using a Biocare Decloaking Chamber (Biocare Medical, Concord, CA) and a pH 6 citrate buffer. Tissue sections were incubated in 3% H_2_O_2_ for 20 minutes to quench endogenous peroxidase activity. GFAP sections were blocked for 30 minutes with 3% normal horse serum (Vector Laboratories, Burlingame, CA), Iba1 and TNF-α sections were blocked for 30 minutes with Biocare Background Punisher (Biocare Medical). Primary antibodies (anti-GFAP, 1:2500 in PBS, or anti-Iba1, 1:100 in TBS, anti-TNF-α, 1:50 in PBS) were incubated for either 2 hours at room temperature or overnight at 4°C, respectively. Negative control sections consisted of primary antibody omission. Next, sections were incubated with an HRP-conjugated anti-rabbit secondary antibody (Vector Laboratories) for 20 minutes followed by aminoethylcarbazole (AEC Solution, Life Technologies, Carlsbad, CA). Slides were counterstained with Gill’s hematoxylin (Richard Allan Scientific, Kalamazoo, MI).

### Image Acquisition and Quantification of Iba1 and GFAP IHC

Seven ROI were selected for microscopic analysis: rostral cortex, striatum, caudal cortex, hippocampus, midbrain, cerebellum, and medulla. These areas were chosen as they represent brain regions previously implicated in CRCI [24–26] and as they demonstrated significant hypometabolism using [^18^F]FDG-PET. From each ROI, three to five adjacent, non-overlapping, 200X magnification images were captured using an upright microscope (Olympus BX53 microscope with Olympus DP73 camera, Olympus America Inc., Center Valley, PA) and analyzed using FIJI (FIJI Is Just ImageJ) software (NIH, Bethesda, Maryland). GFAP^+^ and Iba1^+^ cells were manually quantified using the Cell Counter plug-in. To characterize TNF-α expression, a single 4x magnification image of the hippocampus was evaluated.

### Statistical Analysis

Separate mixed effect models were built using R version 3.1.3 (2015-03-09, R Core Team, 2015) for the two cell types of interest (GFAP^+^ astrocytes, Iba1^+^ microglia). A log of the average cell count [log(Average Count)] was used for analysis. The log(Average Count) was analyzed for each brain region (Region), treatment condition (Treatment), and sacrifice day (Day). The differences in immunoreactivity the cell types of interest were compared in untreated control and irradiated mice. MTX immunoreactivity data was only available at the Day 3 sacrifice time point, and the log of the average number of positive cells for the three cell populations of interest were compared to the mice sacrificed three days post irradiation. Change in FDG uptake was analyzed by single factor ANOVA. Two-sided-paired t-tests assessed differences in FDG uptake and hematological parameters between baseline and follow-up. Correlations between FDG uptake and hematological parameters were evaluated by Pearson's correlation coefficient. All statistical analyses were performed with Microsoft Excel 2010 (Microsoft, Redmond, WA, USA).

## Results

### Results of ^18^F-FDG-PET Imaging

**Fig 2:**
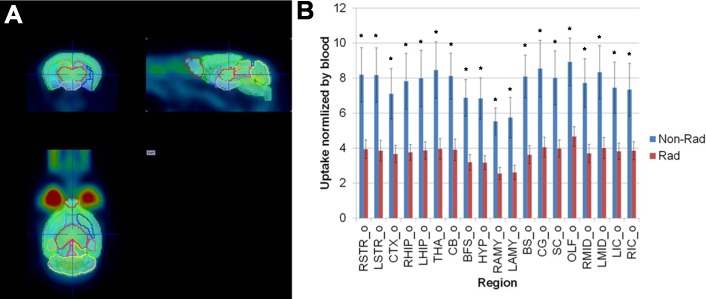
Non-brain directed radiation results in global brain glucose hypometabolism. (A) FDG-PET region of interest (ROI) selection subdivided the mouse brain into 19 areas. (B). When [^18^F]FDG uptake was normalized by blood, significant hypometabolism was noted across all areas, * = p < 0.05. Error bars = standard error of the mean. R/LSTR = Right/Left Striatum; CTX = Cortex; R/LHIP = Right/Left Hippocampus; THA = Thalamus; CB = Cerebellum; BFS = Basal forebrain; HYP = Hypothalamus; R/LAMY = Right/Left Amygdala; BS = Brainstem; CG = Cingulum; SC = Superior colliculi; OLF = Olfactory bulb; R/LMID = Right/Left Midbrain; R/LIC = Right/Left Inferior colliculi.

We first evaluated the effects of NBRT on brain glucose metabolism using [^18^F]FDG-PET. To characterize the sequential effects, mice were examined prior to and 3 days post NBRT. Within the brain, fourteen areas were examined: olfactory bulb, striatum, basal forebrain and septum, cortex, hippocampus, thalamus, hypothalamus, amygdala, cerebellum, brainstem, superior colliculi, inferior colliculi, midbrain, and cingulum. Across all 14 of the selected ROIs, NBRT mice demonstrated significantly decreased (p < 0.05) FDG metabolism as compared to un-irradiated baseline (Fig 2).

### Neuroinflammation in mice administered NBRT or MTX

Next, the brains of mice administered either NBRT or MTX were evaluated for neuroinflammation using anti-microglial (Iba1) and anti-astrocyte (GFAP) IHC. In each brain, seven ROI were selected: the rostral cortex, striatum, caudal cortex, hippocampus, midbrain, cerebellum, and medulla. For the cell types evaluated, no significant differences in cell numbers were observed between CONT mice on day 3 or 30 so they were combined into a single control group (data not shown).

#### NBRT–Day 3

**Fig 3:**
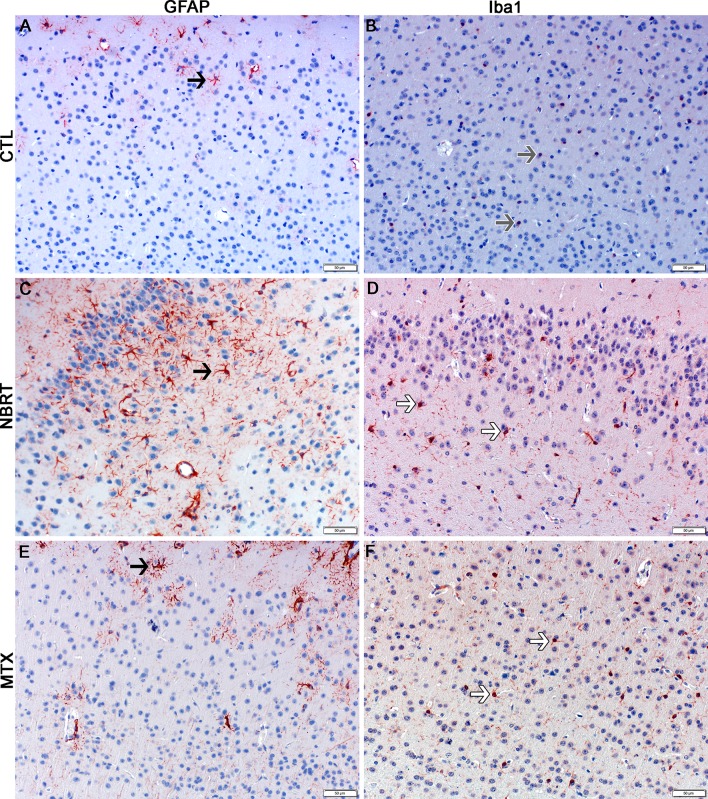
The rostral cortex of NBRT-treated mice reveals early astrocytosis and microgliosis. In the rostral cortex of mice administered NBRT sacrificed at day 3, increased numbers of GFAP^+^ astrocytes (C, black arrow) were identified as compared to CONT mice (A, black arrow). No significant differences in astrocyte numbers were noted when MTX mice (E, black arrow) were compared to the NBRT mice (C). Increased numbers of Iba1^+^ microglia (red, white arrow) (D) were identified in NBRT mice as compared to CONT mice (B, gray arrow). No significant differences were noted when MTX mice (F, white arrow) were compared to the NBRT mice (D, white arrows). In contrast to the small, minimally ramified microglia in the CONT mice (B, gray arrows), the microglia in the NBRT and MTX treated mice demonstrated features consistent with activation (D and F, white arrows).

**Fig 4:**
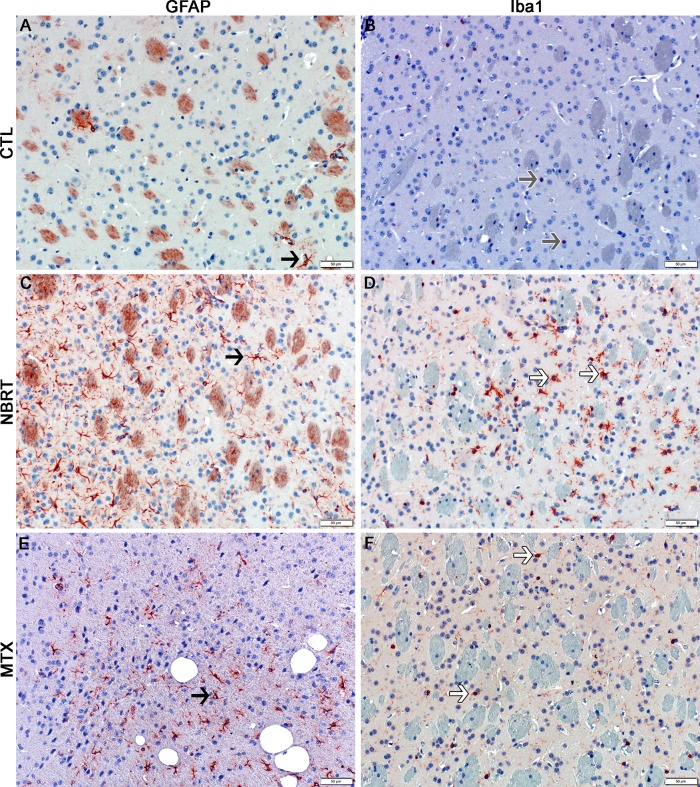
The striatum of NBRT treated mice reveals early astrocytosis and microgliosis. In the striatum of the mice administered NBRT sacrificed at day 3, increased numbers of GFAP^+^ astrocytes (C, black arrow) were identified as compared to CONT mice (A, black arrow). No significant differences in astrocytes were noted when MTX mice (E, black arrow) were compared to the NBRT mice. Increased numbers of Iba1^+^ microglia (D, white arrows) were identified in the NBRT mice as compared to CONT mice (B, gray arrows). No significant differences were noted when MTX mice (F, white arrows) were compared to the NBRT mice (D). In contrast to the small, minimally ramified microglia in the CONT mice (B, gray arrows), the microglia in the NBRT and MTX treated mice demonstrated features consistent with activation (D and F, white arrows).

**Fig 5:**
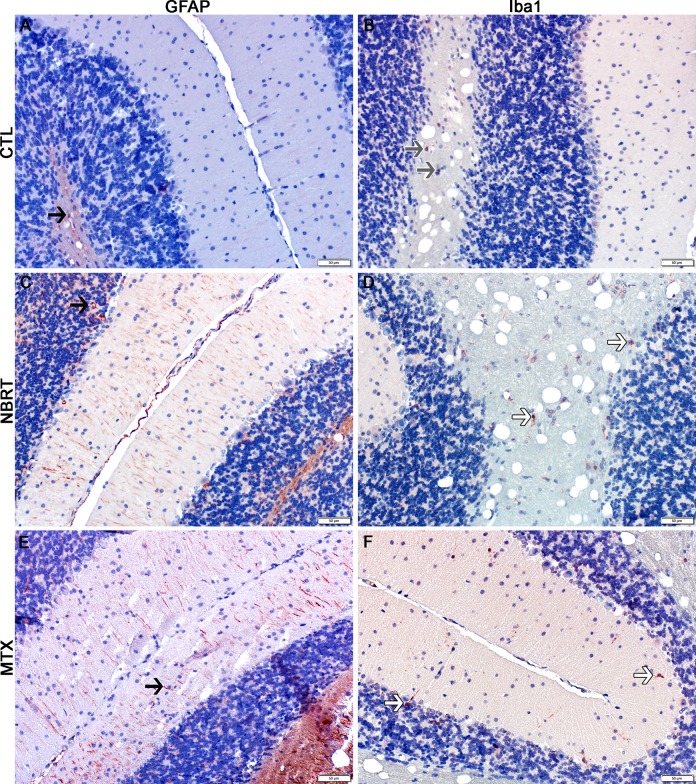
The cerebellum of NBRT treated mice reveals early astrocytosis and microgliosis. In the cerebellum of the mice administered NBRT sacrificed at day 3, increased numbers of GFAP^+^ astrocytes (C, black arrow) were identified as compared to CONT mice (A, black arrow). No significant differences in astrocytes were noted when MTX mice (E, black arrow) were compared to NBRT mice. Increased numbers of Iba1^+^ microglia (D, white arrows) were identified as compared to CONT mice (B, gray arrows). No significant differences were noted when MTX mice (F, white arrows) were compared to the NBRT mice (D). In contrast to the small, minimally ramified microglia in the CONT mice (B, gray arrows), the microglia in the NBRT and MTX treated mice demonstrated features consistent with activation (D and F, white arrows).

**Fig 6:**
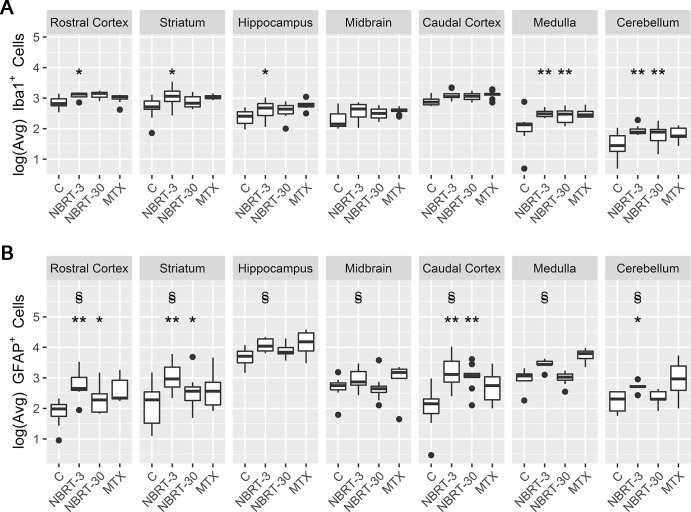
Non-brain directed radiation therapy results in a multifocal, early and persistent astrocytosis and microgliosis. (A) Log (Average) Number of GFAP^+^ cells in NBRT (sacrifice days 3 and 30), MTX, and CONT (C) mice. * = p < 0.05, ** = p < 0.01, compared to controls; § = p < 0.05, compared to NBRT Day 30. (B) Log (Average) Number of Iba1^+^ cells in NBT (sacrifice days 3 and 30), MTX, and CONT (C) mice. * = p < 0.05, ** = p < 0.01, compared to controls; § = p < 0.05, compared to NBRT Day 30.

At day 3 as compared to the CONT mice, the NBRT mice demonstrated increased numbers of GFAP^+^ astrocytes and Iba1^+^ microglia in the rostral cortex, striatum, and cerebellum (Figs 3–6). In addition to increased numbers, the microglia in the NBRT treatment groups commonly demonstrated morphologic features consistent with activation. Specifically, the activated microglia had larger cell bodies and thicker processes than the resting microglia in the CONT mice, which had smaller cell bodies and thinner process [27].

**Fig 7:**
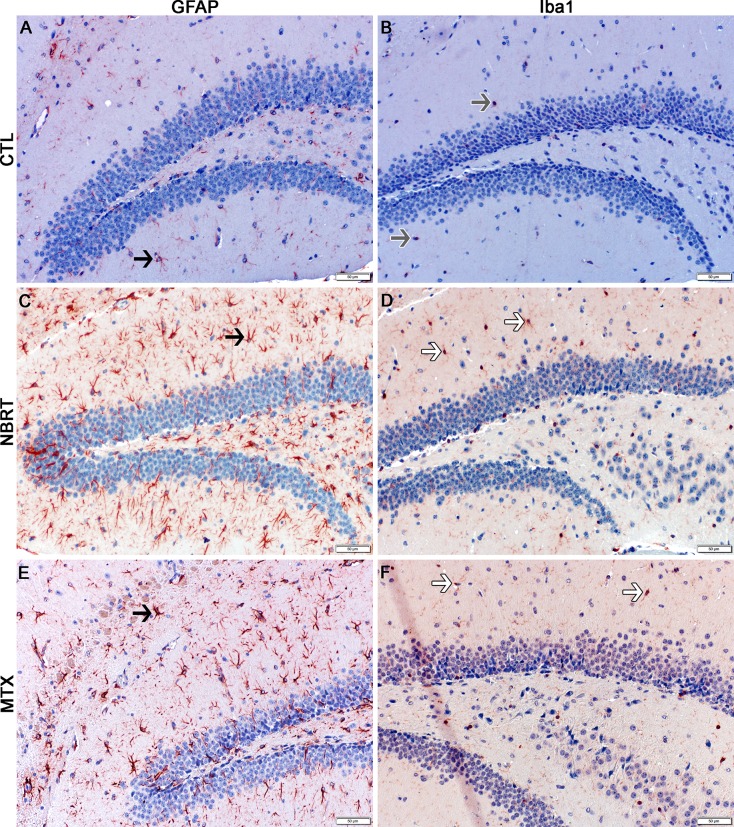
The hippocampus of NBRT treated mice demonstrates early microgliosis, but not astrocytosis. In the hippocampus of the mice administered NBRT sacrificed at day 3, there was no statistical difference in the numbers of GFAP^+^ astrocytes (C, black arrow) as compared to CONT mice (A, black arrow). No significant differences were noted when MTX mice (E, black arrow) were compared to the NBRT mice. Increased numbers of Iba1^+^ microglia were identified in the NBRT mice (D, white arrows) as compared to CONT mice (B, gray arrows). No significant difference in astrocyte numbers were noted when MTX mice (F, white arrows) were compared to NBRT mice (D). In contrast to the small, minimally ramified microglia in the CONT mice (B, gray arrows), the microglia in the NBRT and MTX treated mice demonstrated features consistent with activation (D and F, white arrows).

**Fig 8:**
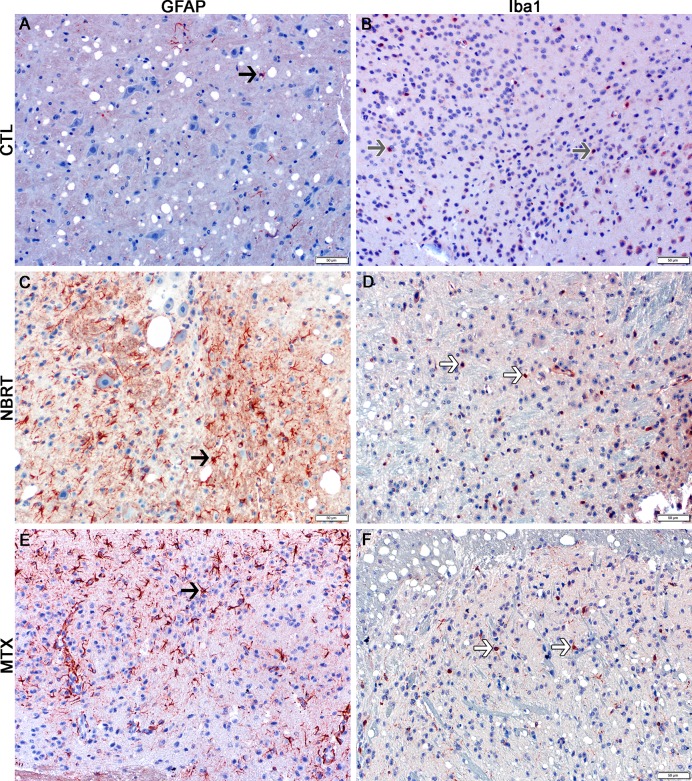
The medulla of NBRT treated mice demonstrates early microgliosis, but not astrocytosis. In the medulla of the mice administered NBRT sacrificed at day 3, there was no statistical difference in the numbers of GFAP^+^ astrocytes (C, black arrow) as compared to CONT mice (A, black arrow). No significant difference in astrocyte numbers were noted when MTX mice (E, black arrow) were compared to the NBRT mice. Increased numbers of Iba1^+^ microglia were identified in the NBRT mice (D, white arrows) as compared to CONT mice (B, gray arrows). No significant differences in microglia numbers were noted when MTX mice (F, white arrows) were compared to NBRT mice (D). No appreciable difference in microglia morphology was noted between the CONT (B, gray arrows), NBRT, and MTX mice (D and F, white arrows).

**Fig 9:**
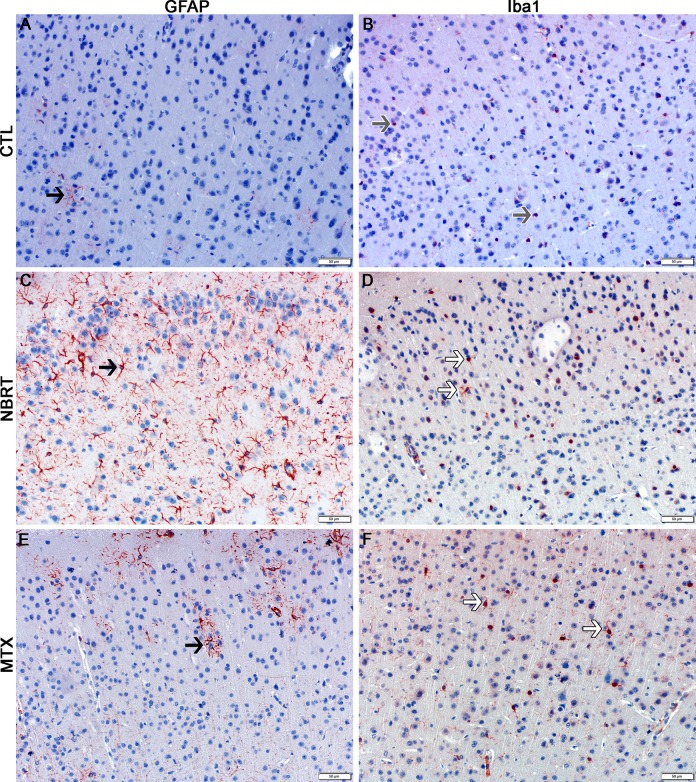
The caudal cortex of NBRT treated mice demonstrates early astrocytosis, but not microgliosis. In the caudal cortex of the mice administered NBRT sacrificed at day 3, increased numbers of GFAP^+^ astrocytes (C, black arrow) were identified as compared to CONT mice (A, black arrow). No significant differences in astrocyte numbers were noted when MTX mice (E) were compared to NBRT mice. Similarly not significant differences in Iba1^+^ microglia (D, white arrows) numbers were identified as compared to CONT mice (B, gray arrow). No significant differences in microglia numbers were noted when MTX mice (F) were compared to the NBRT mice (D). In contrast to the small, minimally ramified microglia in the CONT mice (B, gray arrows), the microglia in the NBRT and MTX treated mice demonstrated features consistent with activation (D and F, white arrows).

In contrast, increased numbers of microglia but not astrocytes were found in the hippocampus (Figs 6 and 7) and medulla (Figs 6 & 8) of NBRT mice as compared to CONT mice whereas only significant astrocytosis was found in the caudal cortex of the NBRT mice (Figs 6 & 9). As in the previous brain regions, the microglia in the NBRT treatment groups demonstrated morphologic features consistent with activation, including larger cell bodies and thicker processes than the resting microglia in the CONT mice.

### MTX–Day 3

We next sought to compare the neuropathology associated with NBRT with that resulting from the administration of MTX, which is well-known neurotoxic chemotherapeutic agent. In comparing the day 3 NBRT mice with the MTX mice sacrificed at day 3, we identified no significant numerical or morphologic differences between the two groups across seven examined brain regions indicating that the neuroinflammatory profile between the two groups was similar (Figs 3–9).

**Fig 10:**
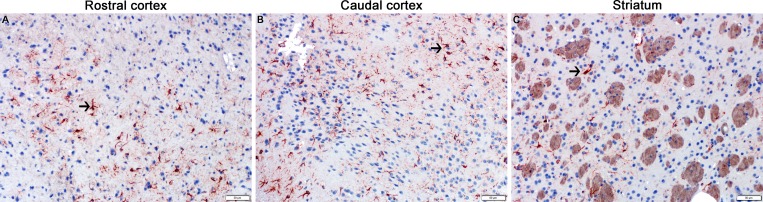
The NBRT treated mice demonstrate persistent multifocal astrocytosis. In NBRT mice sacrificed at day 30, increased numbers of GFAP^+^ astrocytes were identified in the rostral cortex, caudal cortex, and striatum (A-C, black arrows).

When comparing the NBRT mice sacrificed at days 3 and 30, a significant decrease in astrocyte numbers was identified in all seven brain regions with astrocyte numbers returning to near CONT mice in the hippocampus, midbrain, medulla, and cerebellum (Figs 6 & 10).

**Fig 11:**
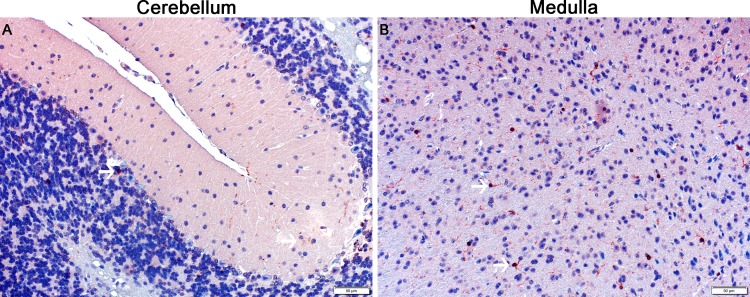
The NBRT treated mice demonstrate persistent multifocal microgliosis. In NBRT mice sacrificed at day 30, increased numbers of Iba1^+^ microglia were identified in the cerebellum and medulla (A-B, white arrows).

As described above, early microgliosis was observed in 5 of 6 brain regions. This increase was persistent to day 30 in the medulla and cerebellum as compared to un-irradiated controls (Figs 6 & 11). Morphologic evidence of microglial activation was only evident with the medulla. Unlike the astrocytes, no significant decreases in microglia numbers were detected when comparing NBRT day 3 with NBRT day 30. While the midbrain was selected as a ROI, there were no significant increases in astrocytes or microglia at either day 3 or day 30 when NBRT mice were compared to CONT.

### TNF-α expression in mice administered NBRT or MTX

**Fig 12:**
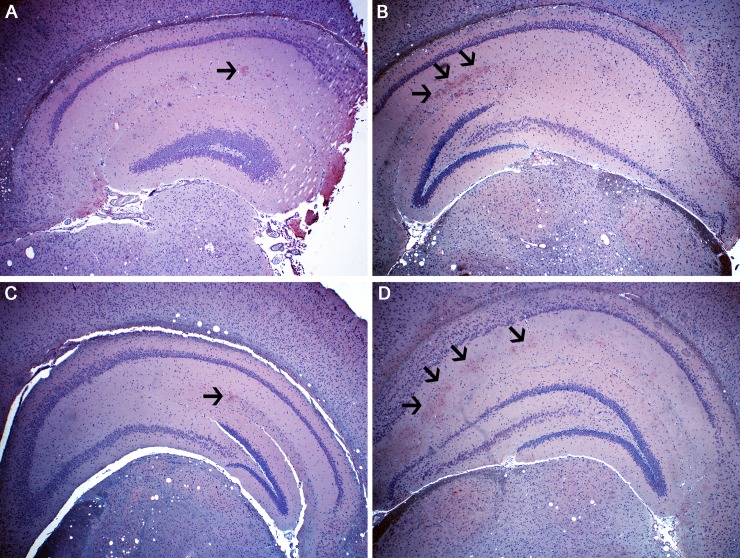
NBRT and MTX mice demonstrate increased hippocampal TNF-α expression. Increased TNF-α immunoreactivity (red) was identified in the hippocampus of NBRT (B, black arrows) and MTX (D, black arrows) mice sacrificed at day 3 as compared to CONT (A, black arrow). In contrast, the level of TNF-α immunoreactivity in mice administered NBRT sacrificed at day 30 (C, black arrow) appears similar to CONT (A).

Following our observations of early and persistent neuroinflammation in the brains of NBRT and MTX mice, we next sought to investigate the role of pro-inflammatory cytokines in the pathogenesis of this phenomenon. As it has been implicated in previous studies of CRCI [4,28,29] and is readily detectable in formalin-fixed, paraffin embedded tissues, we evaluated brains of MTX, NBRT, and CONT mice for TNF-α expression. Owing to its involvement in cognitive impairment in previous rodent studies and its apparent involvement in the NBRT and MTX mice, we chose to focus on the hippocampus. In these initial studies, we revealed increased TNF-α expression in the hippocampus of NBRT and MTX mice as compared to CONT mice (Fig 12). Although limited study material precluded an extensive characterization of the cytokine profile within and between groups, these initial findings support involvement for TNF-α in the brain bystander pathology of NBRT and MTX.

## Discussion

The main goal of this study was to characterize the potential brain bystander effects of NBRT. In summary, we demonstrate for the first time that a single fraction of clinically-relevant NBRT is sufficient to induce significant global brain hypometabolism and multifocal microglial and astrocytic neuroinflammation in mice. Moreover, the neuroinflammation seen in NBRT mice was cytologically and anatomically indistinguishable from MTX-treated mice. Based upon this work we propose that the bystander effects of NBRT on the brain may contribute to the development of CRCI and are worthy of further study.

We initially evaluated the effects of NBRT on brain glucose metabolism using [^18^F]FDG-PET, which is a non-invasive method of assessing neuronal and synaptic activity and, to a lesser degree, glial cell status in neuroinflammatory and neurodegenerative diseases [30]. In the NBRT mice, we identified global brain FDG hypometabolism in mice three days after irradiation. Although the events underlying this change require further study, our findings are similar to those reported in breast cancer patients, in which alterations in FDG metabolism and diminished cognitive function have been described following treatment with radiation and/or chemotherapy [25,31]. The signaling pathways that might mediate the brain bystander effects of radiation in breast cancer patients remain unknown, but there is increasing evidence implicating ROS. Zhang et al report that the administration of ionizing radiation to breast cancer cells results in an increased production of the ROS O_2_^–^ in both directly irradiated and neighboring cells [32]. These findings align with previous work demonstrating that unirradiated bystander cells demonstrate significant oxidative stress when co-cultured with irradiated cells [33]. Moreover, Rockenbach et al provide *in vivo* support for ROS-mediated signaling in work describing that treated human breast cancer patients, 90% of which received radiation, demonstrate significant evidence of circulating oxidative stress as revealed by decreased antioxidant capacity, decreased reduced glutathione and increased concentrations of lipid (thiobarbituric acid reactive substances and lipid hydroperoxides) and protein (carbonyls) oxidative markers [34].

Both the NBRT and MTX treated mice demonstrated significant neuroinflammation as evident by increases in microglia and astrocytes numbers and morphologic evidence of microglia reactivity. Microglia are resident immune effector cells of the CNS and their activation is described in a number of conditions, including inflammatory, infectious, and traumatic disease. Although the end effect of their activation following NBRT requires further study, microglia can adopt either a neurotoxic or neuroprotective phenotype [35]. In the NBRT group, microglial activation was initially widespread, affecting five of seven examined areas. However, by day 30, it had resolved in three of these areas. While the details of the microglial response to NBRT have yet to be intensely studied, acute, transient, and persistent microglial activation has been reported in other brain bystander studies. In lipopolysaccharide (LPS)-treated mice, microglial activation can be detected within 6 hours after treatment and can persist for up to 3 months [36]. Moreover our findings, particularly those revealing hippocampal microgliosis, are similar to those reported in rodent models of chemotherapy-induced CRCI. In rats administered methotrexate, increased numbers of Iba1 positive microglia were identified in the hippocampus and both 1 and 3 weeks post treatment [3]. Similarly, rats treated with cyclophosphamide demonstrated increased numbers of activated microglia (i.e. CD68+ or ED-1+) throughout multiple regions of the hippocampus, including the dentate hilus, dentate gyrus, and the CA1/3 subfields, as compared to sham-treated controls [37,38].

In addition, we identified transient and persistent astrocytosis in the NBRT mice. Astrocytes are cellular residents of the CNS involved in maintaining neuronal function and homeostasis and, like microglia, respond to many CNS insults [39]. While there is no published work examining the impact of NBRT on astrocytes, systemic chemotherapy and peripheral LPS administration have been shown to induce both acute and persistent astrocyte activation [4,40].

While this study was not designed to address the mechanisms underlying the impact of NBRT on the brain, we hypothesize our findings result, in part, from the circulatory extension of radiation-induced inflammatory signals to the CNS. This neuroimmune hypothesis is based upon human and rodent studies illustrating that NBRT results in increased circulating IL-1, IL-6, IL-8, IL-10, TNF-α, and reactive oxygen species [4,29], all of which are capable of crossing the blood-brain barrier to induce CNS inflammation [41]. Most relevant to our work is a previous study demonstrating increased circulating IL-6 in mice administered hindlimb radiation [23]. Our preliminary studies revealing increased TNF-α in the hippocampus of NBRT and MTX treated mice at day 3 supports this hypothesis, but further studies are needed. Ongoing studies seek to characterize the impact of both CT and NBRT on circulating cytokines and markers of oxidative stress. Further support for this mechanism stems from work showing that systemic chemotherapy induces peripheral cytokine production, microglial activation, hippocampal dysfunction, and cognitive impairment [42,43]. Although we hypothesize that the brain bystander effects of NBRT result from the production of pro-inflammatory cytokines and ROS, the exact cellular source of these signaling mediators remains uncertain. While the work cited above implicates tumor cells as the source of ROS and cytokines, it is also likely that peritumoral tissues, including skeletal muscle, participate as well. Notably, previous studies have shown that contracting and radiation-injured skeletal muscle is capable of releasing ROS [44,45]. Finally, although a vascular conduit seems most likely, neural trafficking may contribute, as vagal-mediated signaling can rapidly induce brain cytokine production and neuroinflammation [46].

The limitations of this work include a lack of behavioral data that would provide clinical context to our findings. While microscopy is not a surrogate for clinical phenotype, the similarities in the neuroinflammatory profile between the NBRT mice and other models of cognitive impairment, including our own MTX-treated mice, supports a potential deleterious impact of radiation on cognition [42,47]. Follow-up studies to evaluate the neurobehavioral consequences of NBRT in mice are currently underway. Additionally, we lack measured scattering dose data for tissues outside of the radiation field. While scattered radiation has the potential to impact our interpretations, previous animal studies suggest that the biologic and microscopic effects of this radiation are unlikely to be significant [48]. The results of these studies further expand the *in vivo* scope of radiation induced bystander effects and, despite uncertainty about the clinical consequences of these findings and their mechanisms, we believe these novel findings should prompt further studies on the contribution of NBRT to the initiation and progression of cancer therapy related cognitive impairment.
